# Can we predict the presence of struvite stones based on clinical factors?

**DOI:** 10.31744/einstein_journal/2025AO1324

**Published:** 2025-09-08

**Authors:** Nara Lie Utiyamada, Gabriel Esteves Gaiato, Tamara da Silva Cunha, Reuli Cordeiro da Silva, Felipe Placco Araújo Glina, Alexandre Kyoshi Hidaka, Antonio Corrêa Lopes, Sidney Glina

**Affiliations:** 1 Centro Universitário FMABC Discipline of Urology Santo André SP Brazil Discipline of Urology, Centro Universitário FMABC, Santo André, SP, Brazil.; 2 Universidade Federal do Rio de Janeiro Nephrology Division Rio de Janeiro RJ Brazil Nephrology Division, Universidade Federal do Rio de Janeiro, Rio de Janeiro, RJ, Brazil.

**Keywords:** Struvite, Urinary calculi, Urolithiasis, Crystallography, Ureteroscopy, Signs and symptoms

## Abstract

**Objective:**

This study aimed to evaluate whether clinical, laboratory, and radiological data could effectively identify struvite stones without the need for crystallographic analysis.

**Methods:**

Stone fragments obtained using endourological procedures were subjected to crystallographic analysis. A prospective evaluation and comparison were conducted between patients with and without struvite stones. Sex, age, comorbidities, Hounsfield Unit Coefficient, stone size, urine culture, and urinary pH were analyzed.

**Results:**

Among the 221 stones enrolled, 18% were struvite. Among patients with struvite stones, 95% were women, whereas in the group without struvite stones, 51% were women. The average age was 40.6 years among patients with struvite stones, and 51.5 years in the other group (p<0.001). The stone size in the struvite group (24.9 mm) was significantly larger than that in the non-struvite group (15.6 mm) (p<0.001). The urinary pH was significantly higher in the struvite group than in the non-struvite group (p<0.001). Patients with a positive urine culture had a 3.78 times greater chance of having a struvite stone than those with a negative urine culture (p<0.01). Multivariate analysis considering pH, age, and stone size yielded an AUC value of 0.83, sensitivity of 0.39, specificity of 0.95, and accuracy of 0.85. There was no significant difference between the groups in terms of the Hounsfield Unit Coefficient and comorbidities.

**Conclusion:**

Our analysis further supports the conclusion that characteristics such as pH, age, stone size, and urine culture have notable specificity but low sensitivity for identifying struvite stones.

## Highlights

■Understanding the composition of urinary calculi is essential for guiding long-term treatment strategies.

■This is the first study to correlate clinical data with crystallographic analysis of urinary stones.

■Clinical criteria allow for a high likelihood of accurately identifying stone composition.

■Clinical findings can serve as valuable tools for guiding treatment decisions.

## In Brief

Urolithiasis is one of the major reasons for medical consultations. Accurate identification of the type of urinary stone is essential to ensure appropriate treatment. Understanding the clinical characteristics of patients based on the type of urinary calculi they present with is crucial for guiding medical management and preventing complications.

## Graphical abstract

**Figure:**
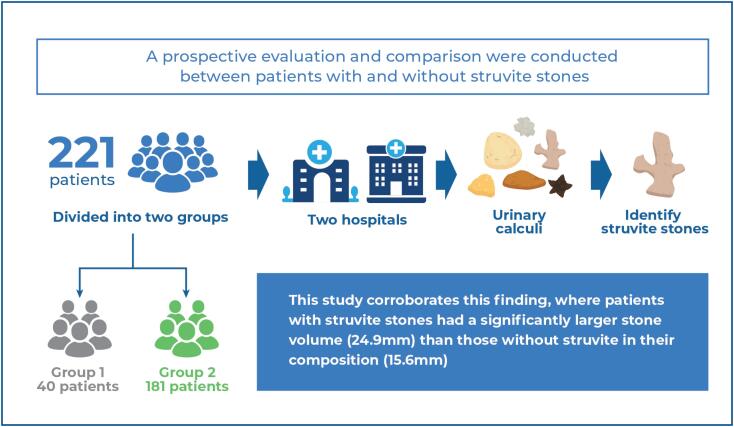

## INTRODUCTION

Recent data have shown that the global prevalence of urolithiasis is 7-11%, and in South America, it is approximately 4%.^(1)^ This variation depends on numerous factors such as social condition, climate, eating habits, and physical activity, and comorbidities such as high blood pressure, diabetes mellitus, and obesity.^(2)^

The recurrence rate reaches 50% within ten years and 26% within five years. Stone composition predicts the risk of recurrence^(3)^ and commonly varies according to sex, age, location, and comorbidities. Calcium oxalate stones are the most common type of stone, with a prevalence of approximately 70%. Struvite stones have a lower prevalence (approximately 5%) globally.^(4)^

Struvite stones are frequently associated with urinary tract infection caused by urease-producing bacteria.^(5)^ They are generally suspected in large and rapidly growing stones, especially in female patients or patients with neurogenic bladders, malformations, or urinary tract obstruction.^(6,7)^

Despite being one of the diseases most frequently encountered in emergency rooms and representing approximately 0.61% of hospitalizations^(8)^ in our country, there is little data available on the prevalence of different types of urinary stones submitted for crystallography, which is considered the gold standard for composition analysis.^(9)^

Among stone analysis methods, chemical analysis is based on qualitative analysis, has many diagnostic flaws, and has been abandoned.^(10)^ Although this method has an estimated accuracy ranging from 40% to 60%,^(11)^ many laboratories worldwide still use it as the only option for determining stone composition. Crystallographic analysis is considered the gold standard and includes morphoconstitutional analysis, followed by quantitative analysis of the material using infrared spectroscopy or X-ray diffraction.^(12)^

## OBJECTIVE

This study sought to evaluate the predominant characteristics of this type of stone and determine the safety of predicting struvite stone composition based on these parameters.

## METHODS

The study included patients aged >18 years who were surgically treated for urolithiasis during the study period and agreed to participate. These procedures were performed at two hospitals affiliated with our college. Patients under 18 years of age and those who did not agree to sign the Informed Consent Form (ICF) were excluded from the study. Stone fragments for which the samples were insufficient for crystallographic analysis were also excluded. Stones removed by endourological procedures were used for crystallographic analysis. All calculi were subjected to morphoconstitutional inspection using an Opton TNG stereoscope, and subsequently to quantitative analysis using an infrared model (Bruker Alpha 2016).

The fragments were sent to the laboratory where one of the researchers, who was unaware of the clinical, laboratory, and radiological images, performed the crystallographic analysis. All calculi were divided into two groups: struvite stones and other compositions. The sex, age, and comorbidities of the patients were recorded. Simple preoperative urinary analysis and culture were performed. The size and density Hounsfield unit (HU) of the stones obtained from the CT scans were recorded. The Shapiro-Wilk test was used to verify the data distribution. Quantitative variables are presented as means, medians, standard deviations, and 25th and 75th percentiles, with 95% confidence intervals. Color, Student's *t-,* and Mann-Whitney tests were performed for comparative data. Stata version 14.0 was used to perform statistical analysis. This study was approved by the Research Ethics Committee (CEP) of *Centro Universitario FMABC* CAAE: 64723722.0.0000.0082:#5.954.836.

Logistic regression analysis was conducted to evaluate the effects of various predictor variables on the outcomes of struvite and non-struvite stones. The variables included in the model were age, urinary pH, stone size, urine hardness (UH) coefficient, urine culture positivity, and diabetes diagnosis. Prior to modeling, we checked the assumptions of multicollinearity using the variance inflation factor (VIF) and tolerance indicators to ensure independence among the explanatory variables.

The benefits of the model were assessed using several criteria, including Deviance, Akaike Information Criterion, Bayesian Information Criterion, and Nagelkerke's Pseudo R². These indicators were also used to compare different models. To measure the predictive capacity of the model, we calculated performance metrics such as accuracy, sensitivity, specificity, and area under the curve (AUC). Additionally, we plotted a receiver operating characteristic (ROC) curve to visually represent the discriminative performance of the model.

All statistical procedures were performed using JAMOVI software, with the significance level set at 5% (p<0.05).

## RESULTS

Two hundred and twenty-one calculi were analyzed, and three were excluded because of an insufficient sample size for analysis. The most prevalent composition was calcium oxalate mono-and dihydrate (65.1%), whereas the lowest was cystine (0.45%) and whitlockite stones (0.45%). Struvite stones accounted for 18.3% (n = 40) of the samples (Table 1).

**Table 1:** Compositions of the analyzed stones

Composition	n (%)
Calcium oxalate dihydrate	71 (32.5)
Calcium oxalate monohydrate	71 (32.5)
Struvite	40 (18.3)
Uric acid	31 (14.2)
Brushita	3 (1.3)
Cystine	1 (0.45)
Whitlockite	1 (0.45)

Group 1 (struvite stones) comprised 95% women, and Group 2 (other compositions) comprised 51% women (p<0.001). The mean age in Group 1 (40.6 years) was significantly lower than that in Group 2 (51.5 years) (p<0.001). Regarding comorbidities, obesity was more common in Group 2, but the difference was not statistically significant (Table 2).

**Table 2:** Analysis of variables in Groups 1 and 2

		Group 1 Struvite n (%)	Group 2 Other n (%)	p value
Sex				<0.001
	Male	2 (5)	88 (49)	
	Female	38 (95)	93 (51)	
Age (SD); years		40.6 (12.6)	51.5 (13.5)	<0.001
Comorbidities		6 (15)	66 (37)	
	Obesity hypertension	5 (12.5)	47 (26.4)	
	Diabetes	3 (7.5)	25 (14)	
	Paraplegia	1 (2.5)	0	
Urinary pH (SD)		6.4 (0.545)	5.9 (0.767)	<0.001
Positive urine culture		11 (27.5)	7 (3.9)	0.019
Size (SD); mm		24.9 (20.5)	15.6 (10.6)	<0.001
HU (SD)		924 (219)	950 (318)	0.10

SD: standard deviation; HU: Hounsfield unit.

On urinalysis, the pH in Group 1 (6.4) was higher than that in Group 2 (5.93) (p<0.001). In addition, those with a positive urine culture had a 3.78 times greater risk of having an infectious stone than those with a previous negative urine culture (p<0.01). Urine culture was positive in 27.5% of patients in Group 1 (Table 3), with the most frequently isolated pathogen being *E. coli* (54.5%), followed by *Proteus* spp. (18%) and *Klebsiella* spp. (18%). In Group 2, the urine culture was negative in 96.1% of the patients. Among the positive cultures, the isolated pathogen was *Klebsiella* spp., followed by *E. coli* (Table 4).

**Table 3:** Most prevalent bacteria in urine cultures of struvite stones

Bacteria	n (%)
Negative	29 (72.5)
*Escherichia coli*	6 (54.5)
*Klebsiella pneumoniae*	2 (18)
*Proteus mirabilis*	2 (18)
*Staphylococcus saprophyticus*	1 (9)

**Table 4:** Most prevalent bacteria in urine cultures of non-struvite stones

Bacteria	n (%)
Negative	174 (96.1)
*Escherichia coli*	3 (1.66)
*Klebsiella pneumoniae*	4 (2.2)

Stone size before the surgical procedure was also a significant factor. Group 1 had larger stones (average, 24.9 mm) than group 2 (15.6 mm) (p<0.001). Tomographic density (HU) was not a significant factor in distinguishing between the groups.

**Figure 1:**
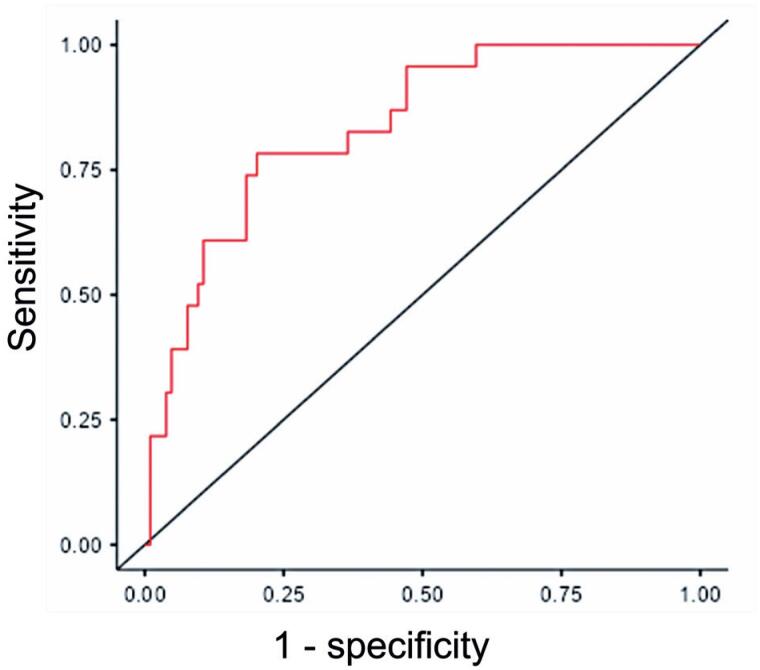
ROC curve of the model including pH, age, and size

Figure 1 shows the sensitivity, specificity, and AUC after multivariable analysis considering pH, age, and stone size. The AUC was 0.83, sensitivity 0.39, specificity 0.95, and accuracy 0.85 (Table 5).

**Table 5:** Coefficients of the struvite model

Predictor	Estimates	Lower limit	Upper limit	Standard error	Z	p	Odds ratio	Odds ratio Lower limit	Odds ratio Upper limit
Intercept	-5.3848	-10.17841	-0.5911	2.4458	-2.2	0.028	0.00459	0.000038	0.554
pH	1.0936	0.35554	1.8316	0.3765	2.9	0.004	2.98487	1.427	6.244
Age	-0.0792	-0.12335	-0.0351	0.0225	-3.52	<0.001	0.92384	0.884	0.966
Size (mm)	0.0422	-0.00264	0.087	0.0229	1.84	0.065	1.04308	0.997	1.091

Controlling for pH and age, the odds ratio (OR) for developing struvite stones increased to 3.04. Conversely, an OR of 0.92 suggests that for every 1 unit increase in age, the odds decrease by 7.47%.

The ROC curve showed that the model using urinary pH, age, and stone size demonstrated high specificity (0.95), but low sensitivity (0.39).

This table presents the effects of pH, age, and size on the risk of struvite and non-struvite stone development. The estimates represent the log odds of "struvite = Yes" *versus* "struvite = No." The combination of these variables had high specificity (0.95) but low sensitivity (0.39) for identifying struvite stones.

## DISCUSSION

In this study population, the prevalence of struvite stones (18%) was higher than the global average of 5%.^(8,9)^ This finding is in accordance with the study by Siener et al., which found a higher prevalence of struvite in countries such as India (23%) and Pakistan (18%), demonstrating that local conditions and the rate of development are involved in the composition of the stones.^(13)^ Struvite stones are more common in women,^(14,15)^ as shown in this study, which could be explained by the higher incidence of urinary infections in the female population.

The average age of patients with struvite stones in Australia is 61 to 70 years,^(16)^ and the average found in a Brazilian study by Danilovic et al. was 48.8±14.9 years,^(17)^ which demonstrates that geographic location and local conditions may be involved in determining stone composition and age at the beginning of its formation. Thus, local conditions may encourage the earlier development of this type of calculus.

Some studies have shown that urine cultures of non-struvite stones are positive in 31% of cases, and in those with struvite components, the urine culture is positive in 90% of cases, with the most common bacteria being *Proteus* spp. (47%). Contrary to these findings, our study showed a low positivity rate of 27.5% in the preoperative urine cultures of patients with struvite stones. Furthermore, the most frequently isolated pathogens differed from those reported previously.^(18,19)^ Analyzing these findings, we believe that the results of these urine cultures were underestimated. Furthermore, the literature shows a difference between midstream urine culture (used in this study) and renal pelvis urinalysis.^(20,21)^ If urine cultures from the renal pelvis had also been collected and evaluated at the time of the procedure, we may have had a higher incidence of positive urine cultures. Finally, in the case of public service, the delay between conducting preoperative examinations and performing the surgical procedure may have led to urine collection after antibiotic therapy, and the urine became temporarily sterile.

Currently, CT is the gold standard for diagnosing lithiasis, with a high sensitivity and specificity of 94 and 97%, respectively.^(22)^ In some studies, the HU coefficient was used to diagnose and predict the degree of hardness and composition of the stone.^(23,24)^ Specifically for struvite stones, HU values vary in the literature, being 790-2143,^(24)^ 862-944^(25)^ and 549-869^(26)^ HU. In our study, the range was 550-1500 HU, with no statistically significant difference between the two groups. Based on these studies, it can be concluded that it is difficult to determine the composition of struvite stones based on HU alone. Despite this, Rodríguez-Plata et al. stated that it is possible to predict the acute composition of a stone through HU density and the width of the region of interest (HU/mm), even in samples with heterogeneous composition.^(22)^ Marchini et al. also used the HU density in their study.^(27)^ Traditionally, struvite stones have large volumes and constitute most staghorn stones.^(28)^ This study corroborates this finding, as patients with struvite stones had a significantly larger stone size (24.9 mm) than those without struvite stones (15.6 mm).

An odds ratio (OR) of 3.04 indicates that for every 1 unit increase in pH, the likelihood of developing struvite stones is approximately 3.04 times greater when age is considered. In percentage terms, this represented a 204% increase in risk. Conversely. An OR of 0.9253 suggested that for every 1 unit increase in age, the odds of developing struvite stones decreased by 7.47%. This outcome provides a tool for advising patients during follow-up visits. The AUC (0.83) demonstrated notable specificity (0.95); however, it had a low sensitivity (0.39) for detecting struvite stones.

The relationship between *E. coli* and struvite stones is not well understood. However, its association with calcium oxalate stone formation is currently being investigated. Research indicates that *E. coli* may enhance the aggregation and adhesion of calcium oxalate through the activation of inflammatory mechanisms. Additionally, exosomes secreted by bacteria that contain proteins, polysaccharides, and lipids may play a role in the formation of infected stones. One study demonstrated that *E. coli* exacerbates calcium oxalate stone formation via a mechanism involving PPK1/flagellin, leading to renal oxidative injury and inflammation.^(29)^ Furthermore, acidic pH increases the pathogenicity of *E. coli* and *Klebsiella pneumoniae*.^(30)^ It is important to note that our small sample size, which included positive urine cultures, may not have been sufficient to establish a definitive association between *E. coli*, acidic pH, and struvite stone formation.

Precise identification of the stone composition greatly assists in the complete treatment of this condition. Similar to how uric acid stones are efficiently dissolved by urine alkalinization, struvite stones deserve attention because small residual stones can quickly develop after surgical treatment. Therefore, complete removal of these stones and infection control are essential.^(31)^ Although the composition analysis of stones can be performed through chemical or physical mechanisms (infrared spectroscopy or diffraction), chemical analysis does not provide reliable results,^(11)^ leaving spectroscopy as the best option.

Unfortunately, this type of examination is scarce in clinical practice. Nevertheless, our results indicate that in this scenario, when we have a young woman with a large stone, alkaline urine, and positive urinary culture, we can treat the patient for infected stones. However, we strongly recommend that efforts should be made to increase the availability of infrared spectroscopy. To the best of our knowledge, this is the first study to correlate stone crystallographic analysis with clinical data and to confirm that the features mentioned above are compatible with this type of stone.

The main limitation of this study was its small sample size. However, we were able to correlate the clinical, radiological, and laboratory findings with crystallographic analysis, which is the gold standard for stone composition. Thus, we believe that our results are consistent despite the small sample size. Future studies with larger sample sizes may provide more robust data and conclusions.

## CONCLUSION

This study confirmed that struvite urinary stones are more common in individuals with larger stones, particularly among young women with higher urinary pH and a greater incidence of urinary infections. This research strengthens the understanding of the characteristics of struvite stones by examining patients diagnosed with these stones using crystallographic analysis. Our statistical analysis further supports the conclusion that, based on the aforementioned characteristics, we can classify stones as struvite calculi and proceed with appropriate treatment.
